# Has the NHS national extended access scheme delivered its policy aims? A case study of two large scale extended access providers

**DOI:** 10.1177/13558196231216657

**Published:** 2023-11-18

**Authors:** Patrick Burch, William Whittaker, Peter Bower, Katherine Checkland

**Affiliations:** 1 PhD Fellow, Centre for Primary Care, 5292 University of Manchester, Manchester, UK; 2 Senior Lecturer, Manchester Centre for Health Economics, 5292 University of Manchester, Manchester, UK; 3 Professor, Centre for Primary Care, 5292 University of Manchester, Manchester, UK

**Keywords:** general practice, health services research, out-of-hours care, continuity of care

## Abstract

**Objectives:**

In 2018, NHS England mandated that all patients in England should be able to access general practice services outside of ordinary hours. While some patients would access additional hours at their own practice, others would need supra-practice level provision – that is, they would be seen in a different location and by a different care team. The policy aim was to enhance patient access to care, with a particular focus on those who work during the day. This study examines (a) how supra-practice level provision of extended access appointments for general medical problems are operationalised and (b) whether the aims of the policy are being met.

**Methods:**

This study presents qualitative comparative case studies of two contrasting service providers offering extended access. The data collected included 30 hours of clinician-patient observations, 25 interviews with staff, managers, and commissioners, 20 interviews with patients, organisational protocols/documentation, and routinely collected appointment data. Thematic analysis ran concurrently with data gathering and facilitated the iterative adaptation of data collection.

**Results:**

Three cross-cutting themes were identified: extended access is being used to bolster a struggling primary care system, extended access provides a different service to in-hours general practice, and it is difficult for extended access to provide seamless care.

**Conclusions:**

Supra-practice access models can provide effective care for most patients with straightforward issues. When ongoing management of complex problems is required, this model of patient care can be problematic.

## Introduction

The most efficient equitable international health systems are those with strong primary care.^1,2^ However, even economically developed countries with well-developed primary care systems face barriers to access.^3^ In England, there is a list-based system of primary care where all patients are registered with a primary care doctor (general or family practitioner) who provides them with medical care and acts as a gatekeeper to specialist services. Due to workforce issues and growing patient demand, there is concern in England about access to primary care doctors.^4,5^

The Prime Minister’s Challenge Fund was set up in 2013 to ‘help improve access to general practice and stimulate innovate ways of providing primary care services’^6(pi)^ in England. Most funded schemes used the money to extend the hours that GP appointments were available. These extra appointments were often carried out away from a patient’s registered practice and by a different doctor. Evaluations noted that the schemes provided additional appointments.^6^ However, there appeared to be an assumption that an appointment away from the practice would be as effective as one within the practice, with limited attention to unintended consequences.

By October 2018, under the policy of ‘extended access’, this scheme had been expanded to cover the entire population of England.^7^ The policy rationale given for extending access to general practice in England has varied over time, and has included:

• Enabling working patients to access general practice at more convenient times.^8^
• Improving declining patient experience of general practice.^9^

There was also an assumption that improving access and patient experience would lead to a reduction in hospital emergency department use.^10^ Importantly, there was an acknowledgement by policy makers that in some circumstances, patients would need to be seen in a different location and by a different care team.^8,9^ For the first time, routine bookable primary care appointments were to be offered outside of a patient’s normal general practice, with a clinician who may not know them or have access to their full record.

The extended access policy had several requirements but left the delivery up to local Clinical Commissioning Groups (CCGs), regional organisations that organised the local delivery of health care for an average of 200,000 patients.^11^ The requirements in 2018 stated that CCGs were to:^9^

• Provide access to pre-bookable and same day appointments to general practice services in the evenings.
• Commission weekend provision of access to pre-bookable and same day appointments.
• Commission a minimum additional 30 min consultation capacity per 1000 patient population per week.
• Ensure effective connection to other system services including access from and to other primary care and general practice services such as urgent care.
• Provide extended hours to the whole population, not targeted solely to one demographic in isolation.

The service requirements for extended access appear to reflect the underlying policy aims – the aim of providing access to routine services is reflected in the requirement that appointments are bookable and not solely for emergencies, and the aim of improving the patient experience is reflected in the capacity requirement.

Policy evaluations of extended access thus far have mostly been limited to evaluations of pilot projects that took place before the COVID-19 pandemic. In areas where extended access operated, the programme effectively increased the total number of primary care appointments available to patients.^6,12^ The demographic of patients using the service was, on average, younger than the population accessing in-hours general practice.^13,14^ However, the take up of appointments in certain areas was limited and there were barriers to access for some patients (e.g. extended access locations were physically located a considerable distance away from some patients).^12,15^ Local evaluations showed that patients using extended access valued the service and were generally happy with it.^12,16^

Evidence also suggested that patients were using the service because of an inability to get an appointment at their registered practice.^12,17^ However, whilst the offering of more primary care appointments could theoretically improve the patient experience of accessing primary care, comparisons of practices offering extended access and no extended access in 2018 showed no evidence of a wider impact.^18^ Research into the effect of after-hours primary care appointments on emergency department use is mixed and there is limited research looking specifically at the impact of the extended access policy.^19^ There is evidence from one area that extended access was associated with reduced attendance at emergency departments with minor conditions.^20^

Existing research has not investigated how extended access is operationalised, which makes it difficult to understand and interpret particular patterns of outcomes. As such, this paper explores the operationalisation of the policy, including how this may impact the effectiveness of the policy in achieving its aims, and discusses the policy’s wider relevance to other health systems.

## Methods

Qualitative comparative case studies of two providers of extended access were carried out, based on a methodological approach described by Stake.^21^ The case was defined as the provision of general practice appointments for general medical problems through extended access. Extended access for services such as nursing, physiotherapy or phlebotomy were excluded. Data were collected by Patrick Burch (PBu), a practising GP who has worked in-hours, out-of-hours, and in extended access. We postulated that if extended access works as an effective extension of general practice, then there should be minimal gaps and differences between the two services. The care received by patients accessing extended access should appear seamless and continuous with that received in in-hours general practice. The experience of receiving patient centred, joined up, seamless care can be referred to as patient experienced continuity.

An often-cited theory of continuity by Haggerty et al. states that continuity is ‘the degree to which a series of discrete healthcare events is experienced as coherent and connected and consistent with the patient’s medical needs and personal context[.]’^22(p1221)^ It then describes three factors which contribute towards continuity:

• Relational continuity – An ongoing relationship between the patient and one (or more than one) provider of health care.
• Informational continuity – Clinicians and patients having appropriate access to information to enable health care.
• Management continuity – the extent to which the approach to health care over time, and potentially between different providers, is responsive, joined up and coherent.

Whilst this theory has been criticised for being overly elaborate^23^ or not reflective of modern health care,^24^ it does aid in understanding the factors that need to be present for a patient to experience seamless care. This theory was used as a sensitising concept.

### Choice of case studies

Two contrasting service providers were recruited, A and B. The organisational features of the two services are given in Table 1. Service A delivered its extended access appointments through five separate sites, known as hubs. The appointments were available to patients from 75 practices. Service B ha six hubs, serving the patients of 37 practices. Compared with B, service A is an older service with more patients, covering a wider area (an entire city), has a higher proportion of non-white patients, has less integration of IT, is based on GP staff, primarily uses face-to-face consultations, triages patients, but clinicians had no option to personally follow up patients they had seen within the service.

**Table 1.** Organisational features of the two case studies.

	Service A	Service B
Operational from	Pre-2018	2019
Area covered	Urban – Entire city (diverse socio-economic composition)	Urban – part of a metropolitan area (diverse socio-economic composition)
Percentage of non-white population	20.9%	9.1%
Number of hubs	5	6
Number of practices	75	37
IT integration	Variable	Good
Predominant clinical staff	GPs	Advanced nurse practitioners
Default appointment type	Face-to-face	Telephone
Patients clinically triaged?	Yes	No
Staff able to follow up within the hub?	No	Yes

### Data collection

Thirty hours of clinician-patient encounters (between September 2021 and January 2022) were observed. This involved observing the extended access clinics of six clinicians at service A and five at B. Detailed field notes were written up within 24 h of the observations.

Formal audio recorded semi-structured interviews, using topic guides, were carried out with patients, clinical staff and managerial/administrative staff. A total of 45 interviews were carried out between October 2021 and December 2022. The characteristics of the interviewees are given in Table 2 (further information on the interviewees is given as S1 and S2 in the online supplement). In the results section, each interviewee is identified by their role and which of the two services that were associated with. The length of the interviews ranged from 10 min to more than an hour. Interviews were audio transcribed using verbatim transcription. There were also multiple informal telephone, face-to-face, and email conversations with staff members that contained rich insights into the working of the services. A small number of documents from both hubs, including organisational protocols and forms to facilitate communication between hubs and practices, were collected between September 2021 and January 2022. Data were collected by PBu. All data were imported into NVivo 12 for analysis.

**Table 2.** Characteristics of interviewees at services A and B.

	Number at service A	Number at service B
Patients	10	10
GPs and advanced nurse practitioners	9	6
Administration staff, managerial staff, clinical leads and commissioners (i.e. CCG staff)	3	7
Totals	22	23

Note. Staff have only been included in one category, but some staff could partially be included in more than one category. For example, hub clinical leads were also involved in the managerial and clinical aspects of the service.

During the study period, some primary care services were affected by restrictions relating to the COVID-19 pandemic. There had been guidance from NHS England that extended access capacity could be repurposed to support the COVID-19 immunisation programme.^25^ At the time of this study, many extended access services were operational again. However, the effects of the pandemic were ongoing. Most general practices were still offering a predominantly telephone first service and services (including extended access) had to incorporate policies for potential COVID-19 cases (i.e. patients with suspected COVID-19 were seen in special designated areas or separate clinics).

### Data analysis

Analysis ran concurrently with data gathering. The use of two different service providers with contrasting organisational features let assumptions and theories generated at one provider be tested on the other. Interviews, field notes, and relevant parts of organisational documents were coded. Data were coded by PBu and KC.

Initial codes included simple descriptive codes (e.g. ‘description of case’, ‘staff co-ordination between service and practice’) and concepts from Haggerty’s continuity theory (e.g. ‘Informational continuity as a barrier’, ‘management continuity’). *A priori* codes were then supplemented with additional codes that were generated from analysis of the data (e.g. ‘perceived misuse of the service’, ‘treating hub patients differently’) ensuring that we captured unexpected phenomena and issues. Emerging findings were discussed regularly at team meetings (PBu, KC, PBo, and WW), and analytic memos used to capture developing themes, theoretical ideas, and develop a second order analysis.

These stages of analysis are analogous to Stake’s stages of data analysis – description, categorical aggregation, establishing patterns and naturalistic generalisations.^21^ Detailed case study reports containing contextual data were created for each provider, alongside a reflexive diary (PBu). Routinely collected appointment data were also analysed.

## Results

The sections below explore four overall topics that arose from the analysis: the design of the service, patient perspectives, staff perspectives, and communication between extended access and other services.

### Service design

Those involved in commissioning and designing the service were cognisant of the policy aim of improving access to core general practice services for all, particularly those who might struggle to attend in office hours:

We try to make sure the offers with extended access reflect core GMS [general medical services]. But a patient who chooses to attend on a Saturday or a Sunday or an evening because that suits their home life, their work/life balance, their responsibilities, then they should be able to go and attend for a routine appointment or have a relatively urgent [appointment]. (Commissioner, B)

At the same time, there was awareness that extended access hub appointments were not the same as having an appointment within a regular practice and would not be ideal for certain types of patients:

This was kind of imposed on us by NHS England and the government to say it should be like an extension of normal hours general practice. I think we have to be sensible, and problems that can’t be dealt with necessarily by somebody who’s not known the patient would be best dealt with by the practice staff if it’s clinically appropriate. (Service manager, A)

Those designing the service had to make certain decisions to prioritise equity of access or whether to target patients that were more appropriate for the service. Service B offered pre-bookable access to patients from all practices without the need for triage when the patient booked an appointment. Service A offered same day only appointments and asked practices to clinically triage patients for appropriateness before booking. They did not specify what was clinically appropriate. However, there was an expectation that patients presenting with some problems would be deemed less suitable and, therefore, potentially excluded from using the service. Both systems were designed with a view to providing safe one-off appointments, where ongoing responsibility for care could be handed back to the patient’s practice.

The hubs were open at varying times and provided different appointment types (e.g. COVID-19 patients, health care assistants, physiotherapy). Appointments for patients with general medical problems were staffed by either GPs or advanced nurse practitioners (ANPs). Service B had a small number of GPs that worked for the service but most appointments for general medical problems were provided by ANPs, whilst service A used GPs only.

Hubs were in existing practices or other purpose-built primary care facilities. Appointments were available on each weekday evening and on Saturdays and Sundays at both providers. The service was designed so that patients would book 15-min appointments through their registered general practice. Practices in service A triaged patients for clinical appropriateness before booking them an appointment at a hub. Service B allowed any patient to book an appointment. In service B clinical staff were able to personally review patients they had seen by making them a follow up appointment. This was not possible in service A.

Both services had practices feeding patients into the hub operating on different clinical IT systems. Service A required hub clinicians to use one of two systems, depending on the system used by the patient’s practice. Service B used one system alone, requiring the practices with a different system to provide an emailed summary of a patients records in advance of a hub appointment.

The degree of integration with other services (e.g. pathology, radiology) varied. Where direct IT integration was lacking, an alternative mechanism involving hub clinical staff and/or service administration staff communicating with practices via phone or electronic means was available. Neither service was able to directly make secondary care referrals or start repeat prescriptions.

### Patient perspectives

Examination of appointment data between June and December 2021 showed 91% of all service A (non-Covid-19 related) GP appointments were booked up and 93% of ANP/GP appointments at service B were booked.

The predominant motivating factor for patients choosing a GP/ANP extended access appointment at both services was a lack of capacity at their registered practice. As one patient said: ‘There were no appointments in our practice, and it was that or nothing’ (Patient, A).

Some patients chose to use the appointments because of convenience, but these were a small minority. In contrast to the policy aim, several patients reported that using extended access was more inconvenient than having an appointment during the day at their registered practice. When asked whether many patients request out-of-hours appointments, one receptionist said:

Maybe not requesting them, not phoning up and saying, ‘Can we have an appointment in the out of hours?’ But when they phone the surgery asking for an appointment, if we haven’t got anything available, then we’ll look in the out of hours for them. (Receptionist, B)

In theory, extended access appointments were equally available to patients at all practices using the service. In reality, appointments were used much more by certain practices, a pattern noted in other evaluations of extended access.^12^ This was most pronounced at B, where of the 2295 general medical access appointments made between July and December 2021, 1747 (76%) were made at four practices (from a total of 37 practices), with 26 practices making appointments for 10 or fewer patients. In service A, of the 13,806 general medical access appointments made between July and December 2021, 3902 (28%) were made with seven practices (from a total of 75 practices). Underlying practice size (i.e. the population registered with a particular practice that were eligible to make extended access appointments) did not adequately account for the differences observed in either service. More details are given as S3 to S6 in the online supplement.

In both services, appointment systems revealed, at times, several patients booked in from the same practice within a few minutes of each other. This suggests that, at certain times, reception staff were booking consecutive patients into extended access appointments because of a lack of available in-practice appointments.

Some practices complained to both services that they found it difficult to access extended access appointments. Staff responsible for running the service were aware of the unbalanced use of appointments. However, they used the appointments system to assist practices struggling to meet high patient demand. As one manager explained:

There are some surgeries that will have really bad days or weeks, and the system helps them out because of the extended access system helps in that. And we’re fairly relaxed about that happening, providing it’s generally done fairly. (Service manager A)

Both extended access services improved patient access by providing appointments for patients who were struggling to access their regular general practice:

Once, they told me I couldn’t get in with my GP… I couldn’t even get dressed at this point. I couldn’t bend in any which way. I couldn’t lift my legs up. So, again, she said we can send you to the hub. You can be seen sooner. So I waited seven or eight days, in absolute agony…. And then I went to see the hub. (Patient, B)

Patients using the hub were generally not concerned about a lack of relational continuity at an extended access appointment. This was either because they felt their issue could be dealt with by any clinician or because, despite often having a desire for continuity, they did not receive relational continuity at their registered practice.

Although the extended access service was often seen as second best to a daytime appointment in their own practice, most patients perceived the service they received through both services to be of a good standard. Some patients deliberately sought out future care from the hub again because they saw it as easier to access than their own GP. Unless they had personal experience otherwise, patients were generally unaware of the limitations of the hubs in dealing with certain clinical issues:

The positives of going to the hub, I would say, is you get to be seen quicker, rather than wait for your own GP, especially with the delays through COVID and staff sicknesses, things like that. So, for the patients, it’s very useful to have that facility available. (Patient, B)

### Staff perspectives

Most of the patient appointments we observed at service A had been booked on the day, triaged by the patient’s registered practice, and ostensibly involved acute or semi-acute medical issues. Service B’s case mix was much more varied, including patients who needed to receive results, patients requiring medication reviews, patients with chronic symptoms, and patients with complex mental health issues. However, despite the system of triage operated at service A, we observed multiple cases that were complex, required follow-up and may have benefited more from a regular in-hours appointment. There were also cases that had not been triaged. One of the reasons behind this were explained by a hub GP who was also a partner in an in-hours practice, as recorded in our clinical observations:

[The GP] reflected that practices are under a lot of pressure and that, although patients are supposed to be clinically triaged prior to being given a hub appointment, this did not always happen. Reflecting on his practice’s use of hub appointments, he said ‘I’m no angel.’ He described that during a busy on-call day, staff had to do anything and everything they could to cope with patient demand. He said that reception staff were sometimes instructed to ‘use their common sense’ and book patients into hub slots without clinical triage. (Notes from clinical observation of a GP, A)

The attitude of clinicians to the care they provided differed between the two services. Most GPs at service A saw their role in the hub as providing a discrete safe one-off episode of care. One GP, when reminded that the government’s aim for the scheme was to extend general practice, replied:

It’s not, is it? It’s providing a different service. I think at the start I was very sceptical as to the value of the service at all, how much it would be used and needed. Now I think it’s needed in terms of just providing extra appointments, but they’ve got to be used appropriately and it’s for stuff that really can be sorted on the day. So, treatment of an acute problem, you know, like something that needs antibiotics … or something like eczema that needs some steroid cream, you know, something that you can treat and advise about follow-up. If it’s something that needs ongoing management and potentially referral, I think the hub isn’t the place to do that. It might provide a bit of information for the patient or a bit of reassurance for the short term or a bit of general advice about, yes, you do need to see your GP about this, but … it’s not managing that problem. (GP, A)

The situation was more nuanced for the ANPs at service B. They would sometimes follow up patients by booking them into future hub slots so that they could provide relational continuity to the patient. During one session observed at service B, 40% of appointments were taken up with follow-ups. As one ANP recalled:

I’m more likely to follow up with people with mental health [issues] because … we’ve built up that little bit of a relationship…I had one recently where I asked, you know, ‘Do you want to go back to doctor or do you want to follow up with me, you know, in a couple of weeks to see how you’re going?’ … [Because] we’d had a really good conversation, she wanted to speak with me, and I think that’s fair enough. (ANP, B)

However, service B’s ANPs also acknowledged that the hub was not always the ideal place for certain types of patients to be seen and that ultimately a patient’s registered practice should take responsibility for their care. The use of follow-up appointments appeared to be driven by feedback the service had received from local practices, complaints that extended access ANPs were passing back work, ANPs feeling that individual patients would benefit from relational continuity in the short to medium term, concern that certain local practices were not coping with demand and would not be able to give timely follow up to patients that required a review, and the personal satisfaction obtained from completing an episode of care. As one ANP said:

The people I choose to follow up myself are the ones where I feel comfortable because I feel that I am able to offer that continuity of care. I think probably the ones I pass back will be the ones … I’ve exhausted what I know now. They need to see a GP because, actually, they’ve got that deeper level of knowledge. (ANP, B)

### Communication between extended access and other services

The policy clearly specifies that arrangements for extended access services must integrate fully with other NHS services. Informational continuity was generally present. Clinicians within the extended access service usually had access to electronic patient notes. Any information recorded during an extended access consultation was saved directly into the record belonging to the patient’s registered practice. Most cases seen in the hubs were acute problems that were unlikely to require further input from a patient's practice or other NHS services. However, we observed multiple cases in both services where co-ordination between services was required or would benefit the patient. There were protocols in place for formally co-ordinating with practices for ‘urgent’ (service A) or ‘important’ (service B) information.

Although systems were in place, the degree to which communication and co-ordination took place differed depending on the clinician a patient saw. When asked whether they had a vision of what that co-ordination facility between the hub and the practice should be like, one commissioner and GP replied:

No, if I’m honest, and I think that decision I suppose will always be down to an individual clinician, won’t it? And I think that is partly where the difference between what a GP would consider to be absolutely important, practice needs to chase up and ensure that this bit is done, versus, perhaps, what an ANP might consider, are probably going to vary. I mean, they vary between GPs, probably even within the GPs at my practice. (Commissioner and GP, B)

When co-ordination between services was potentially required, clinicians in the hubs took a variety of approaches. Some communicated almost all tasks that arose from an appointment directly to practices:

Patient 2’s consultation was a medication review. The hub ANP was unable to reauthorise it because that needed to be done by the patient’s practice. He sent a task to the practice to do this. He marked the task as urgent. ‘So they’ll see it,’ he stated to me after he sent it. (Notes from clinical observation of an ANP, B)

Others very rarely communicated with practices directly and generally let any responsibility for arranging or co-ordinating care rest with the patient:

The GP stated that he only uses the shift report to hand over urgent issues such as two week waits. He says he writes other points of action in the clinical notes. He pointed out that practices will receive a copy of the notes. I then asked him ‘Do you think all practices action what is written in the clinical notes?’ He responded, ‘Probably not. Then it is down for the patient to chase it up.’ (Notes from clinical observation of a GP, A)

Despite attempts to manage communication appropriately, there were sometimes issues created by hubs and practices managing the same patient in an inconsistent or non-complementary manner. In service B, we observed several patients where a hub appointment had been booked, but by the time of the appointment, the issue had been addressed by the patient’s practice.

Differing management of the same condition between a hub and a practice sometimes created problems. There were instances where a hub clinician suggested a referral would be appropriate, but this was not agreed upon by the patient’s practice. This had the potential to create poor management continuity and patients could be caught in between services or clinicians. One patient struggled to understand why a hub clinician recommended she managed her diabetes with tablets but the regular GP she saw only recommended changing her diet.

[My blood sugar levels fluctuate], every time I’ve had blood tests done, ‘cause I have them done about every 6 months. So, the nurse that rang me from the hub, you know, said that she thought I needed to go on the metformin … So, she said she’d ring the surgery, or she’d email them or, you know, suggesting that I go on these metformin. So, … the doctor from the surgery rang me and she said she didn’t think I needed to. So, I just said, ‘Oh, well, the nurse that rang me initially said she thought I did ‘cause it keeps fluctuating.’ So, she said, ‘Well, it’s not fluctuating that much.’ Well, I’m not going to argue between two professionals. (Patient, B)

## Discussion

This study examined the operationalisation of extended access services and whether extended access is meeting its policy goal of extending access to general practice. Three principal themes were identified through the data: extended access is being used to bolster a struggling primary care system, extended access provides a different service to in-hours general practice, and it is difficult for extended access to provide seamless care.

Regarding the first theme, the original aim of the extended access policy in England was to provide patients with more convenient access to general practice, with a focus upon working people who struggled to attend appointments during the day. This was to have the effect of improving patient experience of general practice. Our results suggest that the policy is only partially meeting its aims. But, in common with previous evaluations,^12,17^ our study found that most patients seen in the hubs were not seeking care outside of normal working hours, they simply wanted an appointment. Hub appointments were used when practices could not provide timely access and were supporting a struggling primary care system.

Extended access appointments were primarily made for patients from particular practices, with some suggestion that these may have been the practices that were struggling most with access issues. Regardless of any potential ethical implications, the extended access policy highlights the practical difficulties of trying to implement policies to increase patient choice in an overburdened health service. As appears to be occurring in extended access, any extra service or capacity will be potentially used up, not out of choice, but out of necessity. A service, designed to facilitate choice and patient convenience, may not be appropriately designed to cater for what it is used for in reality.

Regarding the second theme, extended access offers patients access to additional appointments, but our study suggests that it does not offer access to the full experience of personalised generalist care that is associated with in-hours general practice.^2^ Extended access can deal effectively with acute non-complex problems, but it can struggle to deal with more complex patients who require ongoing care. These inefficiencies can cause delays in decision making, duplication of appointments, additional workload for practices, and increased treatment burden for patients. Despite the efforts of service providers, supra-practice level extended access cannot fully substitute for a properly functioning general practice.

Regarding the third theme, despite generally having access to patients’ medical records, extended access clinicians often struggled to provide or enable joined-up seamless care to patients who required follow up or further care after their appointment. This was partly due to extended access being a service designed for one-off encounters but also due to clinician behaviour and limitations in the co-ordination mechanisms between extended access and in-hours practices.

While there is no evidence that extended access has improved patient satisfaction,^18^ our study found, in common with other evaluations, that most patients that used extended access found the service generally effective in dealing with their needs.^12,17^ However, this positive view of the service must be looked at in the context that most patients had been booked into extended access due to an inability to have a timely daytime appointment at their regular practice. It is not extended access per se that patients value, but the ability to get *any* timely appointment.

One of the broader issues this research raises is whether primary care capacity should be increased through existing general/family practices or whether larger organisations that operate at a supra-practice level should provide this care. If supra-practice level organisations are providing more care, given the known association between relational continuity and better health outcomes,^26–28^ should they be trying to provide relational continuity to patients (i.e. allowing clinicians to follow up patients within the service)?

Since this research was carried out, the specifications required for extended access in England have changed.^29^ They are more flexible than the original specifications and state, amongst other things, that appointments could be for emergency issues only or geared to providing specific services such as smears or physiotherapy. Whether it is better to use supra-practice level care (in this case, extended access) for clearly specified issues, rather than general primary care, requires further research.

## Limitations

There are two main limitations with this study. First, it is based on only two case studies. Additional case studies would have further improved the trustworthiness of the findings. It would have been useful to study a smaller and/or more rural provider of extended access. However, given national pressures and the reorganisation of primary care in England, the main findings of the study are likely to apply across a range of service providers.

Second, this study did not consider non-general medical treatment (e.g. physiotherapy or practice nurse appointments). This was to keep the research within manageable limits. However, a significant proportion of extended access appointments are for such treatment. Future research could consider that element of the service.

## Conclusion

In certain countries, the at-scale provision of primary care appears to be increasing.^30^ Whether this is alongside, or as a replacement for, traditional models of general practice remains to be seen. When it comes to the provision of appointments for general medical problems, supra-practice access models (such as extended access services) may have a role to play in improving access for certain patient groups. They can provide effective care for most patients with straightforward issues, but when ongoing management of complex problems is required, this model of patient care can be problematic. Triage of patients using extended access can mitigate some of these issues but are by no means 100% effective. Increased pressure for appointments on practices can lead to the circumventing of systems designed to ensure the effective use of externally booked appointments.

There have been several proposed solutions to improving access to primary care in England.^31^ One idea is to assign patients to within-practice or supra-practice level primary care based on their demographic and/or clinical characteristics.^32^ Our study suggests that this may be difficult to achieve that in practice, and it may not deliver the increased efficiency required.

## Supplemental Material

Supplemental Material - Has the NHS national extended access scheme delivered its policy aims? A case study of two large scale extended access providers Supplemental Material for Has the NHS national extended access scheme delivered its policy aims? A case study of two large scale extended access providers by Patrick Burch, William Whittaker, Peter Bower, and Katherine Checkland in Journal of Health Services Research & Policy
