# Supplementary material for: Has the NHS national extended access scheme delivered its policy aims? A case study of two large scale extended access providers
Source: J Health Serv Res Policy. 2023 Nov 18;29(3):191–200. doi: 10.1177/13558196231216657 (PMC11151702; doi:10.1177/13558196231216657)

Online Supplement

S1: Characteristics of patients interviewed.

Service	A		B	
Sex	Male<br>Female	3<br>7	Male<br>Female	6<br>4
Chronic health condition?	8		5	
In work	4		5	
Age	18-29<br>30-39<br>40-49<br>50-59<br>60-69<br>70+	0<br>0<br>2<br>2<br>6<br>0	18-29<br>30-39<br>40-49<br>50-59<br>60-69<br>70+	1<br>2<br>2<br>0<br>4<br>1
**Total participants**	**10**		**10**	

S2: Characteristics of clinical staff interviewed.

Service	A		B	
Sex	Male<br>Female	4<br>4	Male<br>Female	0<br>4
Frequency of extended access work	≥ Once a week<br>< Once a week	6<br>2	≥ Once a week<br>< Once a week	3<br>1
Qualified in current role	< 3 years<br>3-10 years<br>>10 years	2<br>1<br>5	< 3 years<br>3-10 years<br>>10 years	1<br>3<br>0
Worked for extended access service	<6 months<br>>6 months	1<br>7	<6 months<br>>6 months	0<br>4
Also works in in-hours general practice	Yes<br>No	6<br>2	Yes<br>No	4<br>0
**Total participants**	**8 (GPs)**		**4 (ANPs)**	

S3: Number of general practice extended access appointments made by individual practices in service A, July-December 2021

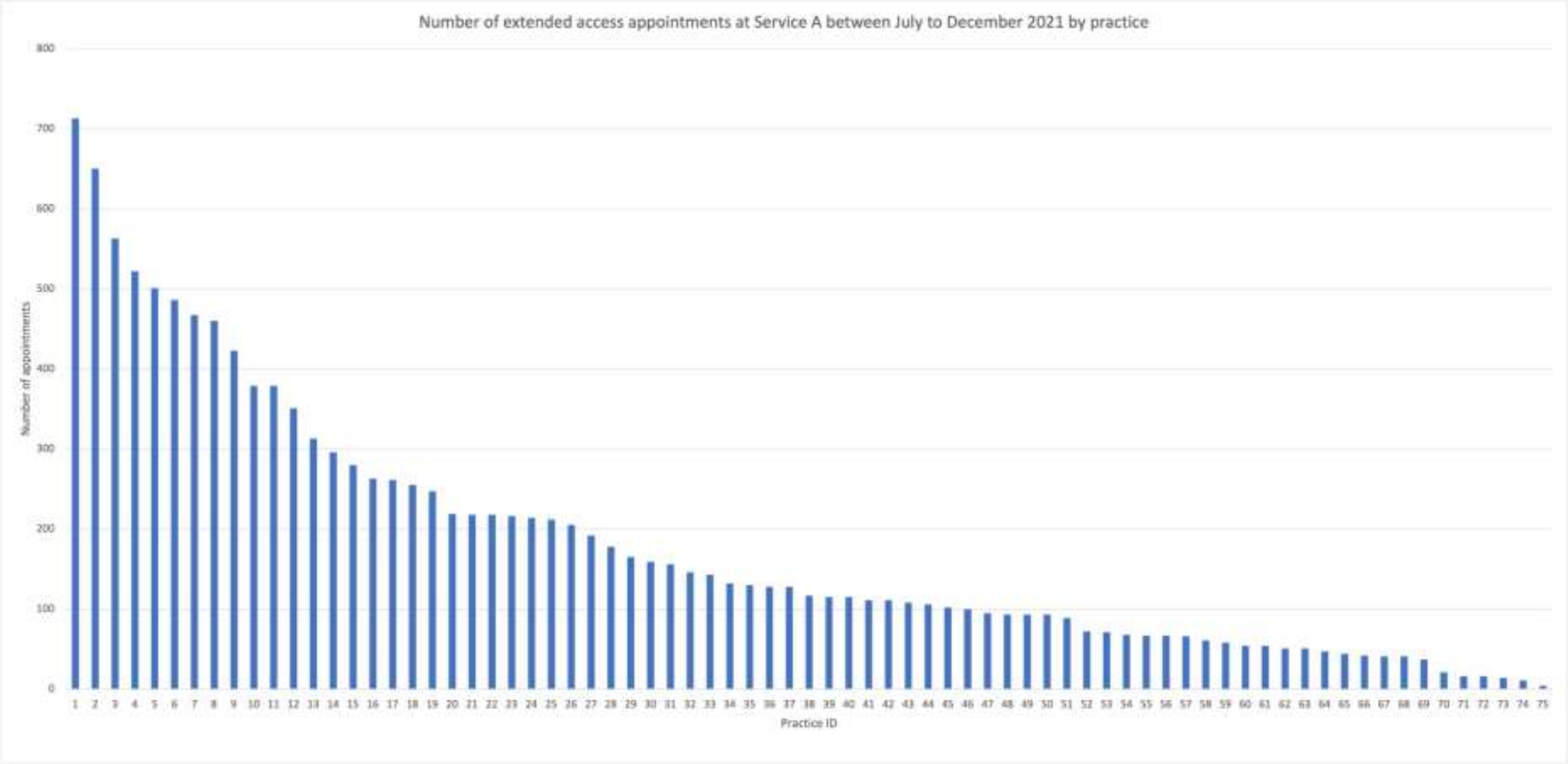

S4: Number of general practice extended access appointments made by individual practices in service B, July-December 2021

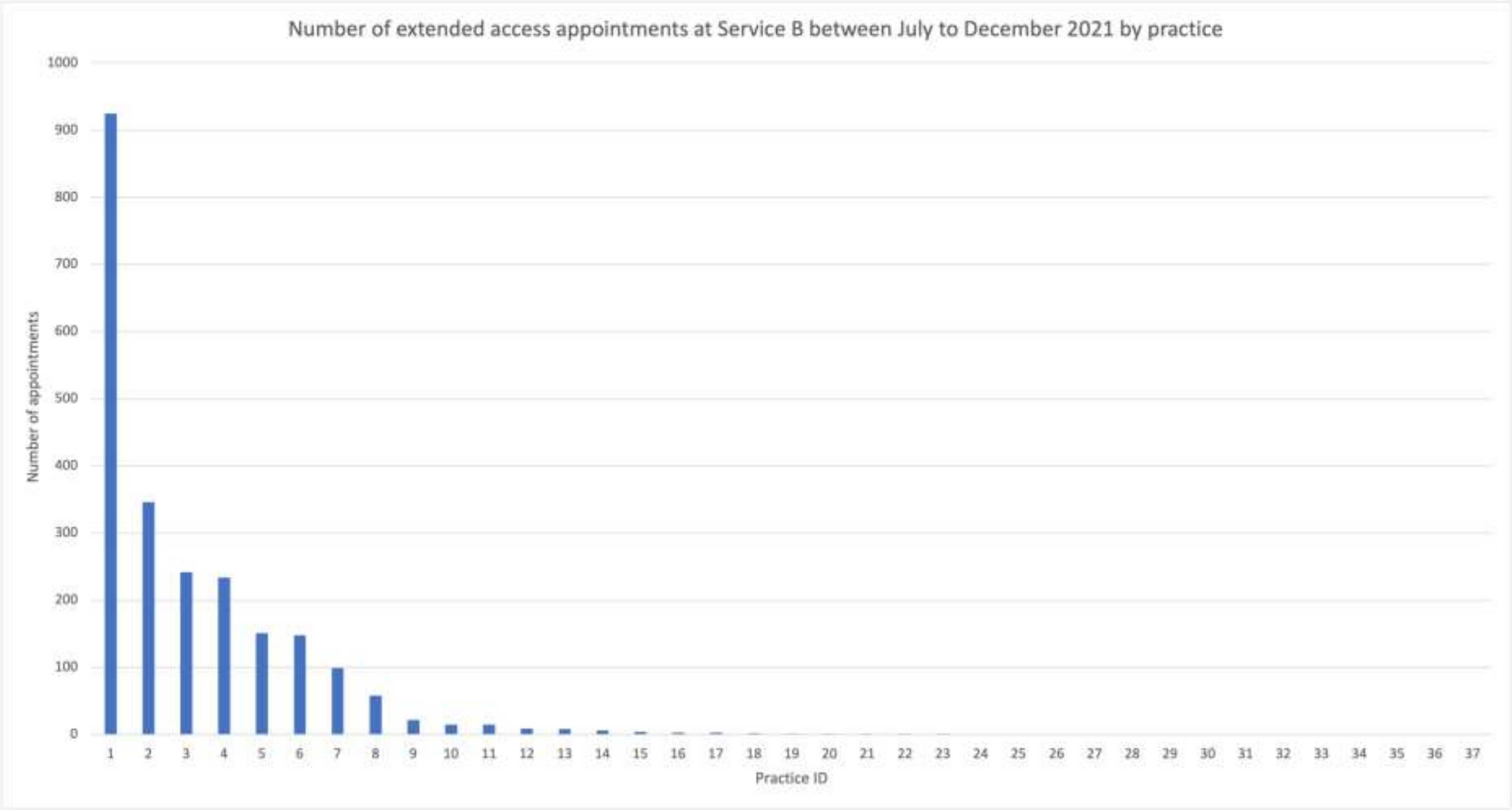

S5: Number of extended access appointments adjusted for practice size in service A, July-December 2021

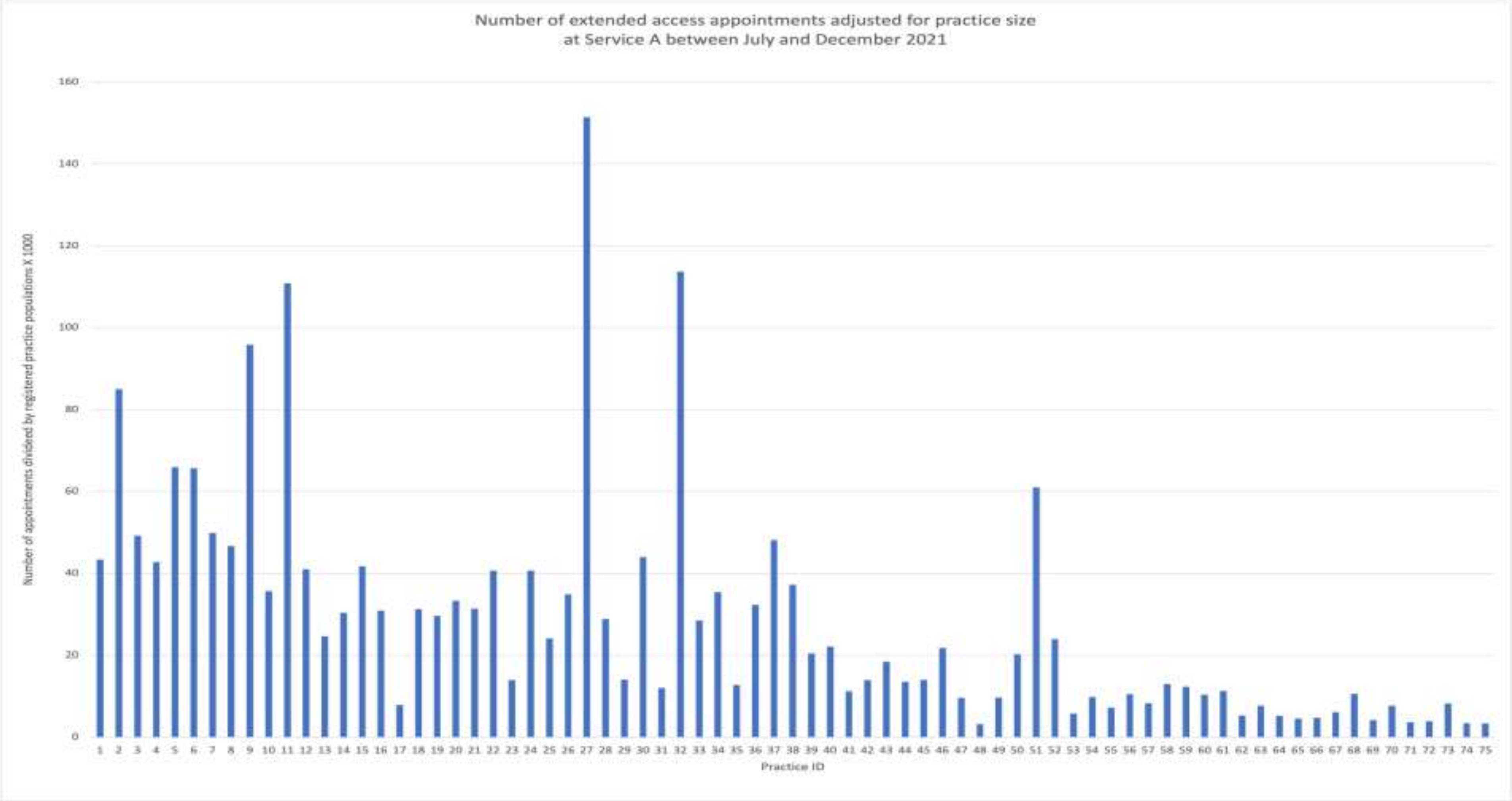

S6: Number of extended access appointments adjusted for practice size in service B, July-December 2021

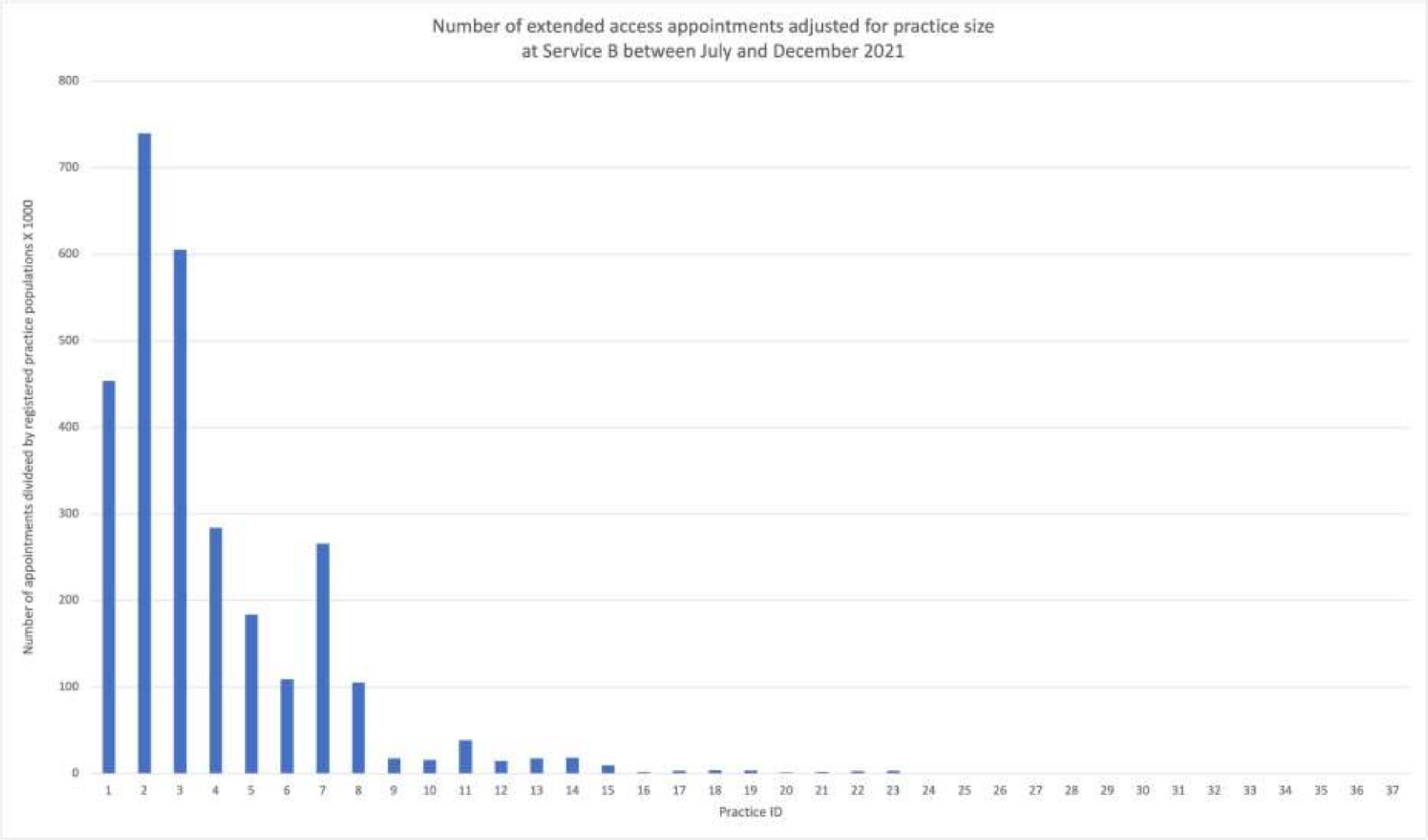

Supplement: Supplemental Material - Has the NHS national extended access scheme delivered its policy aims? A case study of two large scale extended access providers [file sj-pdf-1-hsr-10.1177_13558196231216657.pdf]
